# Case Report: Primary clear cell adenocarcinoma of the urethra—imaging features and literature review

**DOI:** 10.3389/fonc.2026.1708651

**Published:** 2026-02-24

**Authors:** Li Yang, Yan Long, Gang Zhou, Ling Wu, Kaichuang Deng, Shuixian Huang, Zheng Xiong

**Affiliations:** 1 Department of Radiology, Gejiu People’s Hospital, Gejiu, Yunnan, China; 2 Department of Ultrasound, Gejiu People’s Hospital, Gejiu, Yunnan, China

**Keywords:** case report, clear cell adenocarcinoma, diagnosis, diffusion-weighted imaging, magnetic resonance imaging, urethral neoplasms

## Abstract

**Background:**

Primary clear cell adenocarcinoma of the urethra (PCCAU) is an extremely rare malignant tumor. Its clinical manifestations are nonspecific, and preoperative diagnosis relies primarily on imaging studies. To date, most reports on this disease are isolated case presentations, and systematic studies correlating imaging findings with pathological results remain scarce.

**Case presentation:**

A 58-year-old woman was admitted due to voiding dysfunction for 14 months, worsened with gross hematuria for 2 months. Preoperative imaging evaluation included ultrasonography (US), computed tomography (CT), and magnetic resonance imaging (MRI). US revealed an irregular hypoechoic mass in the post-bladder urethral region with minimal internal blood flow; CT scan indicated a heterogeneously enhancing mass in the urethral area accompanied by necrotic changes; MRI demonstrated a periurethral lesion showing a slightly high signal on T2-weighted imaging (T2WI) with a low-signal capsule. Diffusion-weighted imaging (DWI) showed restricted diffusion, with an apparent diffusion coefficient (ADC) value of 0.79 × 10^-^³ mm²/s, heterogeneous enhancement in the arterial phase of dynamic contrast-enhanced imaging, and a centripetal filling pattern. Postoperative histopathology confirmed the diagnosis of PCCAU. Immunohistochemical findings were as follows: AE1/AE3 (+), CK7 (focal +), PAX8 (+), P504S (+), Napsin A (scant +), p53 (~5%+), and Ki-67 (~35%+). The patient declined adjuvant therapy after surgery. Seven months postoperatively, she developed lymph node metastasis and peristomal metastasis around the right ureteral abdominal wall orifice, and was subsequently treated with toripalimab immunotherapy. She remained alive at the 1-year postoperative follow-up.

**Conclusions:**

PCCAU is extremely rare. Imaging examinations, particularly multimodal MRI, combined with histopathological and immunohistochemical analyses, play a crucial role in its diagnosis. Early surgical intervention contributes to improved prognosis, and personalized treatment strategies, including immunotherapy, may offer clinical benefits in advanced cases.

## Background

Primary clear cell adenocarcinoma of the urethra (PCCAU) is an exceedingly rare malignant tumor of the urinary system, accounting for less than 0.003% of all urethral malignancies (1). Its histogenesis remains controversial, with proposed origins including mesonephric duct remnants, Müllerian derivation, and urothelial differentiation (2). Histologically, the tumor is characterized by clear cytoplasm, hobnail cells, and nuclear positivity for PAX-8, resembling carcinomas of Müllerian origin (3, 4). Clinical manifestations are nonspecific; common symptoms such as hematuria, dysuria, and a urethral mass can mimic other urethral disorders, often leading to misdiagnosis (4, 5). Consequently, imaging evaluation becomes crucial for preoperative diagnosis, although current evidence is largely confined to case reports. This article presents a case of PCCAU evaluated with multimodality imaging, including 3.0 T magnetic resonance imaging (MRI), contrast-enhanced computed tomography (CT), and ultrasonography (US). By integrating these imaging findings with a review of the literature, we aim to summarize the characteristic imaging features of PCCAU.

## Case presentation

**Figure 1:**
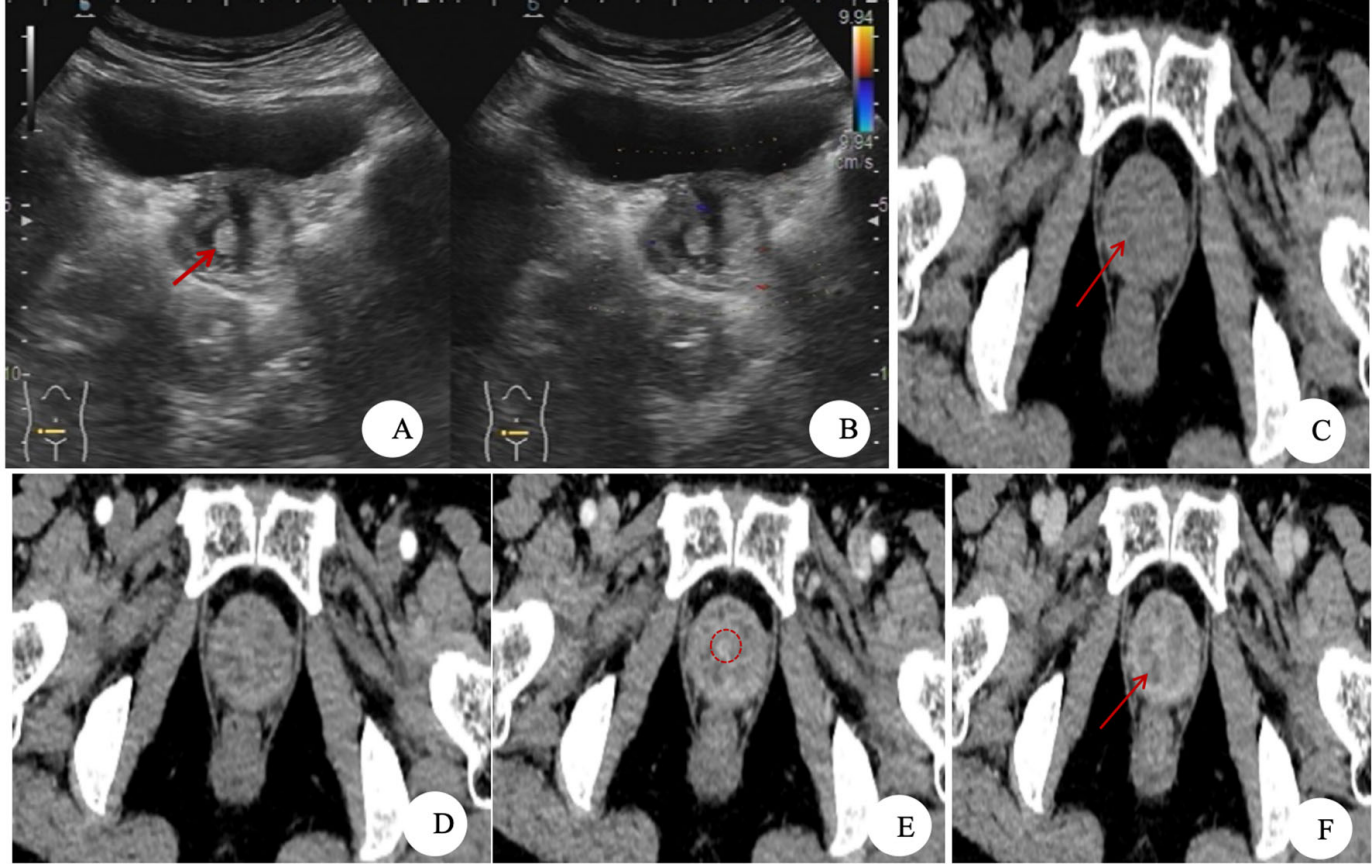
Images of clear cell adenocarcinoma of the urethra (CCAU). **(A,B)** Ultrasonographic images. **(A)** An irregular mass with ill-defined margins is observed in the post-bladder urethral region, demonstrating heterogeneous echogenicity. A small hyperechoic area (red arrow) and an anechoic zone are visible within the mass. **(B)** Color Doppler flow imaging (CDFI) demonstrates scant blood flow signals within the mass. **(C–F)** CT images. **(C)** Non-contrast CT: A soft tissue density mass is observed in the posteroinferior urethral region to the bladder, with partially ill-defined margins. Patchy areas of low density (red arrow) are noted within the mass. **(D–F)** Contrast-enhanced CT, arterial, venous, and delayed phases: The mass demonstrates marked heterogeneous enhancement. Patchy non-enhancing necrotic areas (red arrow) and a circumferentially enhancing urethral image (red dashed circle) are visible within the mass.

**Figure 2:**
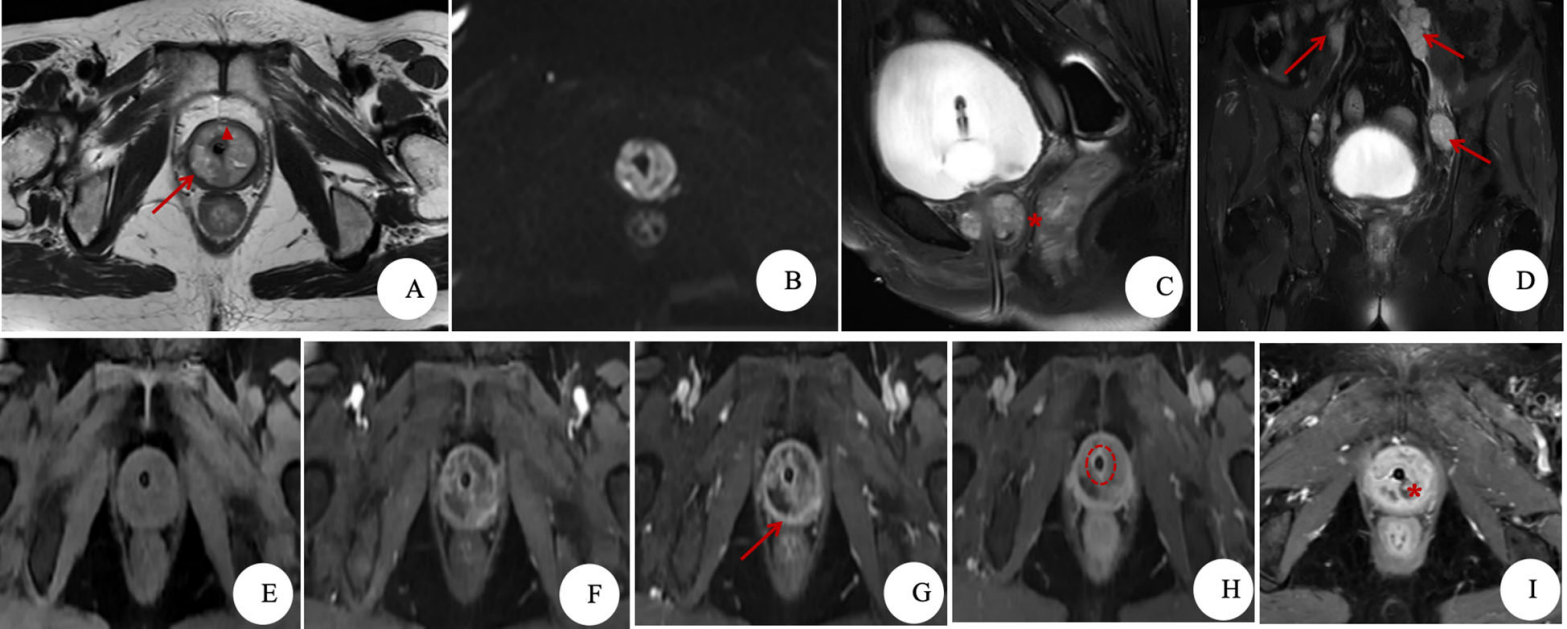
MRI findings of clear cell adenocarcinoma of the urethra (CCAU). **(A)** Axial T2WI: An annular, round-like mass is visible around the upper urethra, demonstrating slightly high signal intensity (compared to muscle). Within the mass, small patchy areas of high signal and punctate low signal foci (red triangle) are observed, surrounded by a low-signal capsule (red arrow) with well-defined margins. **(B)** DWI, *b* = 1,000 s/mm²: The mass shows restricted diffusion, with an ADC value of 0.79 × 10^-^³ mm²/s. **(C)** Sagittal T2WI: The mass exhibits clear boundaries with the bladder and vagina (red star), with posterior displacement of the vaginal wall. **(D)** Coronal T2WI: Multiple enlarged lymph nodes are visible along the bilateral iliac vessels (red arrow). **(E)** Pre-contrast mask: The mass shows iso- to slightly low signal intensity (compared to muscle). **(F–I)** DCE series: The tumor is hypervascular and demonstrates progressive heterogeneous enhancement. Non-enhanced necrotic areas are noted within the mass (red triangle), with prominent capsular enhancement (red arrow). Circumferential enhancement of the urethral wall is visible on certain sequences (red dashed circle), and the local urethral morphology appears irregular (red star).

1. Patient information: A female patient (58 years old) was admitted due to voiding abnormalities for 14 months, aggravated with gross hematuria for 2 months. The patient initially presented with straining to void, urinary frequency, urgency, and dysuria without obvious cause 14 months ago. Two months prior to admission, she developed painless, terminal gross hematuria accompanied by blood clots. No abdominal pain, vaginal bleeding, or abnormal discharge was reported. The patient was previously healthy and denied a family history of genitourinary or other malignancies.
2. Physical examination: An oval-shaped mass was palpable along the anterior vaginal wall, which was non-tender. Pressure applied to the mass resulted in bloody discharge from the external urethral meatus.
3. Laboratory investigations
  3.1. Total prostate-specific antigen (PSA): <0.09 ng/mL, free PSA: <0.04 ng/mL. The decision to measure PSA was guided by MRI findings suggestive of a periurethral adenocarcinoma (detailed in Section 4.3). Although elevated PSA is uncommon in female patients, its aberrant expression has been documented in certain adenocarcinomas, including some primary periurethral tumors, thus providing a potential diagnostic clue.
  3.2. Urinalysis: Leukocyte esterase positive, nitrite positive, protein positive, occult blood (3+), and red blood cells (4+). All other parameters were within normal limits.
  3.3. Tumor markers (CA19-9, CA-125, AFP, and CEA) were within normal limits, and the remaining laboratory tests showed no significant abnormalities.
4. Imaging findings
  4.1. US
    4.1.1. US was performed via a transabdominal approach with a 2.5-MHz convex low-frequency transducer.
    4.1.2. US findings (as shown in **Figures 1A, B**): A hypoechoic mass was identified in the post-bladder urethral region, exhibiting ill-defined margins and heterogeneous echogenicity. Within the mass, small hyperechoic areas and anechoic zones were observed. Color Doppler flow imaging (CDFI) demonstrated scant blood flow signals, with pulsed-wave (PW) Doppler revealing a low-velocity, high-resistance arterial spectrum.
  4.2. CT
    4.2.1. CT was performed using a Canon Aquilion ONE GENESIS 320-slice CT scanner. The scan parameters were as follows: tube voltage, 120 kV; automatic tube current modulation was employed. For contrast-enhanced imaging, iohexol (350 mgI/mL) was administered as an intravenous bolus via a power injector at a dose of 1.5 mL/kg and a flow rate of 3.0 mL/s.
    4.2.2. CT findings (as shown in **Figures 1C–F**): Non-contrast CT revealed a soft tissue density mass in the posteroinferior urethral region to the bladder, showing heterogeneous attenuation with an approximate CT value of 38.80 Hounsfield units (HU) in its solid components. Patchy hypodense areas were noted within the mass, with no evidence of calcification. Following contrast administration, the lesion demonstrated progressive, marked heterogeneous enhancement, with the solid portions measuring approximately 52.20 HU in the arterial phase, 67.40 HU in the venous phase, and 78.40 HU in the delayed phase. Non-enhancing necrotic areas were observed internally. On certain imaging levels, a circumferentially enhancing urethral structure was visible at the center of the mass.
  4.3. MRI
    4.3.1. MRI was performed using a United Imaging 3.0 T scanner. The scanning sequences and key parameters were as follows: T2-weighted imaging [T2WI; repetition time (TR) 2,696 ms, echo time (TE) 122 ms, and slice thickness (ST) 3.0 mm], diffusion-weighted imaging (DWI; TR 3,100 ms, TE 0.34 ms, and ST 3.0 mm), and T1-weighted imaging (T1WI; TR 3.36 ms, TE 1.5 ms, and ST 3.0 mm). For contrast-enhanced imaging, gadoteric acid meglumine was administered as an intravenous bolus at a dose of 0.1 mmol/kg, followed by multiphase T1WI.
    4.3.2. MRI findings (as shown in **Figure 2**)
      4.3.2.1 Axial T2WI displayed an annular, round-like mass surrounding the upper urethra, demonstrating slightly hyperintense signal (compared to muscle). The lesion contained small hyperintense necrotic foci and central punctate hypointense signals. A hypointense capsular rim was noted along the periphery, with well-defined margins. The mass measured approximately 3.4 cm (transverse) × 3.2 cm (anteroposterior).
      4.3.2.2 DWI (*b* = 1,000 s/mm²) showed restricted diffusion, with an apparent diffusion coefficient (ADC) value of 0.79 × 10^-^³ mm²/s.
      4.3.2.3 Sagittal T2WI revealed clear demarcation between the mass, bladder, and vagina, with posterior displacement of the vaginal wall. The height of the tumor (the longest diameter parallel to the urethra) measures approximately 2.5 cm.
      4.3.2.4 Coronal T2WI identified multiple enlarged lymph nodes along the bilateral iliac vessels, the largest measuring approximately 1.8 cm × 2.3 cm.
      4.3.2.5 Dynamic contrast-enhanced (DCE) MRI indicated that the tumor was hypervascular and exhibited progressive heterogeneous enhancement. Non-enhanced necrotic regions were noted within the mass. Circumferential enhancement of the urethral wall was visible on selected sequences, associated with local irregularity of the urethral contour. Prominent enhancement of the peripheral capsule was also observed.
    4.3.3. Imaging impression: The findings are initially suggestive of periurethral adenocarcinoma.
5. Cystoscopic findings: The urethral and bladder mucosa were smooth, with no diverticula or intraluminal masses observed. These findings suggest no invasion of the urethral or bladder mucosa by the tumor.
6. Treatment and follow-up

Prior to surgery, the patient underwent a needle biopsy of the periurethral mass at our institution. The pathology was further reviewed by experts from our institution and the Department of Pathology at Peking Union Medical College Hospital, with findings suggestive of metastatic clear cell carcinoma (CCC). The patient subsequently underwent laparoscopic radical cystourethrectomy. The procedure was combined with hysterectomy and bilateral salpingo-oophorectomy, partial vaginectomy, bilateral cutaneous ureterostomy, and pelvic lymph node dissection.

**Figure 3:**
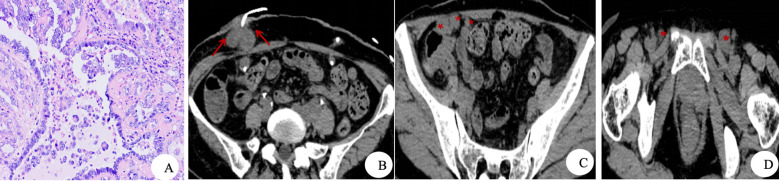
Diagnostic histology and post-treatment follow-up imaging. **(A)** H&E staining, ×400: Tumor cells exhibiting clear cytoplasm and prominent nucleoli are observed. **(B–D)** Non-contrast CT images obtained 7 months postoperatively demonstrate a soft tissue mass adjacent to the right ureteral abdominal wall stoma (red arrow), along with enlarged lymph nodes in the pelvic cavity and bilateral inguinal regions (red star).

Examination of the gross surgical specimen revealed a mass located 0.4 cm from the bladder orifice. The cut surface was grayish-white, solid, and friable, with focal areas of necrosis. Histopathological examination confirmed the diagnosis of PCCAU (as shown in **Figure 3A**). The tumor was found to invade the urethral muscular layer, the muscular layer of the anterior vaginal wall, and the detrusor muscle of the bladder. Lymphovascular invasion was present, and metastatic carcinoma was identified in 7 out of 21 resected pelvic lymph nodes, all surgical margins (urethral, bilateral ureteral, and vaginal) were negative, and the uterus and bilateral adnexa were free of tumor involvement.

Immunohistochemical staining results were as follows: Positive markers: AE1/AE3 (+), CK7 (focal +), PAX8 (+), P504S (+), Napsin A (scant +), p53 (~5%+), and Ki-67 (~35%+). Negative markers: GATA3, CK5/6, p63, ER, NKX3.1, PSA, and WT1.

Postoperatively, the patient declined adjuvant therapy. Seven months later, metastases were identified in the pelvis, bilateral inguinal lymph nodes, and around the right cutaneous ureterostomy (as shown in **Figures 3B–D**). Subsequent genetic testing showed PD-L1 expression [combined positive score (CPS) = 20%], suggesting potential responsiveness to PD-1/PD-L1 inhibitors. Given the histological diagnosis of PCCAU and the patient’s explicit refusal of conventional chemotherapy, toripalimab was initiated for immunotherapy after thorough informed consent and agreement to self-funding, with consideration of drug accessibility, costs, and the patient’s financial capacity. At 1-year postoperative follow-up, the patient remains alive.

**Table 1** outlines the chronology of key clinical events from initial presentation through treatment and follow-up.

**Table 1:** Timeline of diagnosis, treatment, and follow-up in the present case.

Time points (T0 = day of surgery)	Clinical stage	Key events and outcomes
T−14 months	Symptom onset	Voiding abnormalities, specifically manifested as straining to void, urinary frequency, urgency, and dysuria.
T−2 months	Symptom progression	Presented with painless, terminal gross hematuria accompanied by blood clots.
T−2 weeks to T0	Preoperative evaluation	· Imaging findings (US/CT/MRI): A space-occupying lesion was identified in the periurethral region. · Underwent a needle biopsy of the periurethral mass: The mass was consistent with metastatic clear cell carcinoma (CCC).
T0	Surgery	· Histopathological examination: The diagnosis was primary clear cell adenocarcinoma of the urethra (PCCAU), exhibiting muscular invasion (urethra, vagina, and bladder), lymphovascular invasion, pelvic lymph node metastasis, and negative surgical margins. · Postoperative course: The patient declined adjuvant therapy following surgery.
T+7 months	Tumor recurrence	Follow-up CT revealed metastatic disease involving the pelvis, bilateral inguinal lymph nodes, and the area surrounding the right cutaneous ureterostomy.
T+7.5 months	Immunotherapy initiation	· Genetic testing: High PD-L1 expression was detected. · Treatment decision: The patient refused chemotherapy and received toripalimab for immunotherapy.
T+12 months	Follow-up	At 1-year postoperative follow-up, the patient was alive.

## Discussion

Konnakkol et al. first reported clear cell adenocarcinoma of the urethra (CCAU) in 1973. Its histogenesis remains controversial, primarily revolving around origins from the mesonephric duct, Müllerian duct, or differentiation from renal/urothelial lineages (2). A significant association exists between CCAU and urethral diverticula, with approximately 56% of patients with CCAU having a concurrent urethral diverticulum (4). Imaging examinations, including US, CT, and MRI, are useful for determining the tumor’s location, size, morphology, relationship with surrounding tissues, involvement of the bladder or vagina, and presence of pelvic lymph node involvement or distant metastasis. However, definitive diagnosis ultimately relies on pathological examination.

Current reports on this disease focus primarily on clinical and pathological aspects, with imaging studies being relatively scarce. Based on the present case and previous literature reports, we summarize the imaging characteristics of CCAU in **Table 2**: (1) US, serving as an initial screening tool, often demonstrates a hypoechoic or mixed-echoic mass in the urethral region, but its utility is limited for assessing early lesions and depth of infiltration (9). (2) CT is helpful for evaluating pelvic lymph node involvement or distant metastasis. The tumor typically presents as a soft tissue mass in the posteroinferior urethral region to the bladder or as circumferential urethral wall thickening, frequently exhibiting heterogeneous density, progressive marked heterogeneous enhancement, and internal necrosis (5, 10–14). In our case, the CT findings corroborated the imaging features reported previously. (3) Multi-parametric MRI (T2WI, DWI, and contrast-enhanced) can clearly depict the internal architecture of the tumor (e.g., necrosis, septations, and capsule) and its biological behavior (4–6, 9, 11, 13–15). In our case, a central punctate hypointense focus was observed on axial T2WI, and the normal target sign (6) of the urethra was partially preserved. These findings are consistent with the report by Takeuchi et al. (14) and indicate predominant submucosal tumor involvement. Kim et al. (15) compared six cases of CCAU with nine non-clear cell adenocarcinoma of the urethra (NCCAU) cases and proposed four imaging characteristic features of CCAU: a lower height-to-width ratio (CCAU: 0.74–1.09 compared to NCCAU: 1.07–1.93), frequent association with urethral diverticulum, common presence of internal septations, and greater preservation of normal urethral architecture. In our case, the tumor demonstrated a height-to-width ratio of approximately 0.74, internal septations, and residual urethral structures, aligning with these features. In addition to validating previously reported features, this study contributes the first quantified ADC value (0.79 × 10^-^³ mm²/s) for this rare tumor and a systematic description of its dynamic enhancement, characterized by strong peripheral enhancement in the arterial phase and progressive centripetal filling in subsequent phases. These insights add objective, quantitative support to imaging diagnosis, pending confirmation in larger studies.

**Table 2:** Summary of imaging features of clear cell adenocarcinoma of the urethra (CCAU).

Category	Imaging findings	Pathologic/Anatomic basis	Reference
Tumor location	· Female: Mostly proximal urethra. · Male: Prostatic urethra. · 56% located within urethral diverticulum (4).	Skene’s glands, Müllerian duct remnants	(4–8)
Ultrasonography	Hypoechoic mass encircling urethra, heterogeneous echotexture, abundant peripheral vascularity on Doppler.	–	(9)
CT	· A round or oval mass around the urethra. · Soft tissue density, mostly heterogeneous in density. · Heterogeneous enhancement with necrotic areas. · An intact/disrupted capsule is visualized.	· Coexistence of multiple components (clear cells, fibrosis, necrosis). · Hypervascular nature.	(5, 10–14)
MRI	**·** T2WI: Predominantly high signal intensity (compared to muscle) with internal low-signal fibrous septa, a peripheral low-signal capsule, and a central punctate low-signal focus (residual urethra). The normal urethral target sign^a^ is intact/partially preserved. · DWI: high signal intensity. · Heterogeneous or rim enhancement. · An intact/disrupted capsule is visualized.	· Glycogen → water-binding → T2 prolongation → T2 hyperintensity. · May relate to high tumor cellularity and compact cellular architecture.	(4–6, 9, 11, 13–15).
Features of our case	· Tumor’s height-to-width ratio^b^ = 0.74. · A central punctate hypointensity (residual urethra) is present within the lesion on T2WI. · ADC = 0.79 × 10^-^³ mm²/s. · DCE (CT**/**MRI): · Arterial phase: Prominent rim enhancement. · Venous/Delayed phase: Centripetal (filling-in) enhancement. · Delayed phase: Heterogeneous enhancement. · The capsule demonstrates marked enhancement and appears disrupted. · Necrosis is present within the tumor, with no evidence of calcification.	· Height-to-width ratio: lower in CCAU (0.74–1.09) vs. NCCAU (1.07–1.93) (Kim et al.) (15).	(15)

a. Urethral target sign (6): On axial T2WI, MRI demonstrates the characteristic layered architecture of the normal urethra, which presents as three concentric rings. From inner to outer, these are as follows: A central hypointense band, corresponding to the urethral mucosa and the urine within the lumen; a middle hyperintense band, corresponding to the highly vascular, gland-rich loose connective tissue of the urethral submucosa; and an outer hypointense ring, corresponding to the dense smooth muscle bundles of the urethral muscularis propria.

b. Height-to-width ratio: the height of the tumor on sagittal T2WI (the longest diameter parallel to the urethra) and the width of the primary tumor on sagittal T2WI (the longest diameter perpendicular to the height of the primary tumor) (15).

c. Key innovation:

· Our study is the first to quantitatively report an ADC value of 0.79 × 10^-^³ mm²/s.

· Describe its dynamic contrast-enhanced enhancement pattern.

Histologically, CCAU may exhibit tubulocystic, papillary, or diffuse growth patterns (3). Its immunophenotype is characteristic: the tumor typically shows positivity for PAX-2, PAX-8, epithelial membrane antigen (EMA), CK (AE1/AE3), CAM 5.2, CK7, AMACR, and p53, while it is negative for GATA3, p63, ER, PR, CK20, and CA-125. The Ki-67 proliferation index demonstrates considerable heterogeneity (3). In our case, P504S expression was positive, aligning with the findings of Sun et al. (16). In their study, all seven cases of primary urinary tract clear cell adenocarcinoma (five bladder, two urethra) were P504S positive, with strong expression in all three cases that had pelvic lymph node metastasis. The positive expression of P504S supports a cloacal/mesonephric-related origin of clear cell adenocarcinoma (CCA). Sun et al. further confirmed that P504S is a highly sensitive marker (sensitivity 100%) for urinary tract CCA, and its expression is not limited to tumors of prostatic origin. Although CCAU is histomorphologically difficult to distinguish from CCC of the female genital tract (4), primary urethral origin was established in this case through systematic exclusion of other sites (no primary lesion detected by imaging in the uterus, ovaries, or kidneys) and its characteristic immunoprofile. Differentiation between primary and secondary CCA requires integration of clinical history and radiological assessment.

CCAU is highly aggressive and carries a poor prognosis with a low 5-year survival rate (4). For patients without distant metastasis, surgery remains the mainstay of treatment, while radiotherapy and chemotherapy can be considered for those with metastatic disease. Patel et al. (17) demonstrated that surgical intervention can improve both overall survival (OS) and disease-specific survival (DSS); however, a standard surgical treatment protocol is still lacking. Genetic testing may provide guidance for precision chemotherapy in CCAU.

In recent years, immunotherapy has emerged as an important therapeutic approach for advanced solid tumors. Immune checkpoint inhibitors targeting PD-1/PD-L1 have become standard treatment for various solid tumors, including clear cell renal cell carcinoma (18) and urothelial carcinoma (1, 19), with their use rapidly expanding from advanced disease to early-stage adjuvant and perioperative settings (20). However, experience with immunotherapy in rare tumors such as CCAU remains extremely limited, primarily based on case reports (21, 22). In our case, the PD-1 inhibitor toripalimab was selected for postoperative recurrence based on two main considerations: the tumor’s morphological similarity to immunotherapy-sensitive CCC and the presence of PD-L1 high expression, which provided a molecular rationale. This treatment represents an individualized approach in the absence of a standard regimen and in the context of the patient’s refusal of chemotherapy. The outcome offers preliminary insight into immunotherapy for this rare tumor type, though its efficacy requires validation in larger studies. Moving forward, biomarker-guided precision therapy will be an important direction for future exploration.

This study has several limitations. First, as a single-center case report, the generalizability of its conclusions is inherently limited. Second, the postoperative follow-up period is currently only 1 year, which is insufficient for evaluating long-term outcomes. Furthermore, although detailed imaging data are provided, the lack of quantitative comparisons with similar cases limits the validation of the imaging features’ generalizability. Finally, the discussion regarding the efficacy of PD-1 inhibitors is based primarily on this case and a limited number of other reports, and thus, their therapeutic benefit requires confirmation from more extensive clinical data. Nevertheless, the systematic multimodal imaging documentation in this case provides a valuable reference for diagnosing this rare disease.

## Conclusion

CCAU is an exceedingly rare malignancy. A multimodal diagnostic approach incorporating imaging studies alongside histopathological and immunohistochemical analysis is essential for achieving an accurate diagnosis. Early surgical intervention may improve patient prognosis.

## Data Availability

The original contributions presented in the study are included in the article/**Supplementary Material**. Further inquiries can be directed to the corresponding author.
