# Supplementary material for: Case Report: Primary clear cell adenocarcinoma of the urethra—imaging features and literature review
Source: Front Oncol. 2026 Feb 24;16:1708651. doi: 10.3389/fonc.2026.1708651 (PMC12971443; doi:10.3389/fonc.2026.1708651)
Supplement: Supplementary file 1 [file DataSheet1.docx]

Supplementary Material

## Supplementary Figures


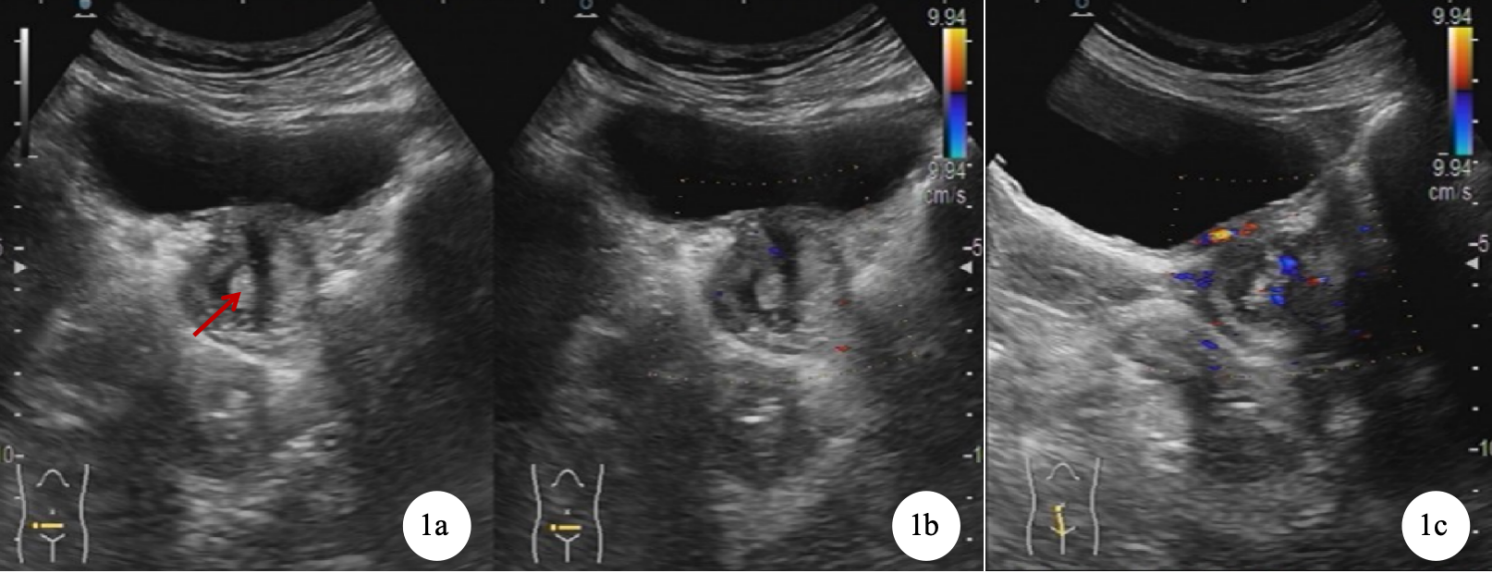


**Supplementary Figure 1. **Ultrasonographic images of clear cell adenocarcinoma of the urethra (CCAU)****

1. **An irregular mass with ill-defined margins is observed in the post-bladder urethral region, demonstrating heterogeneous echogenicity. A small hyperechoic area (red arrow) and an anechoic zone are visible within the mass.(b, c).Color Doppler flow imaging(CDFI): demonstrates scant blood flow signals within the mass.**

**
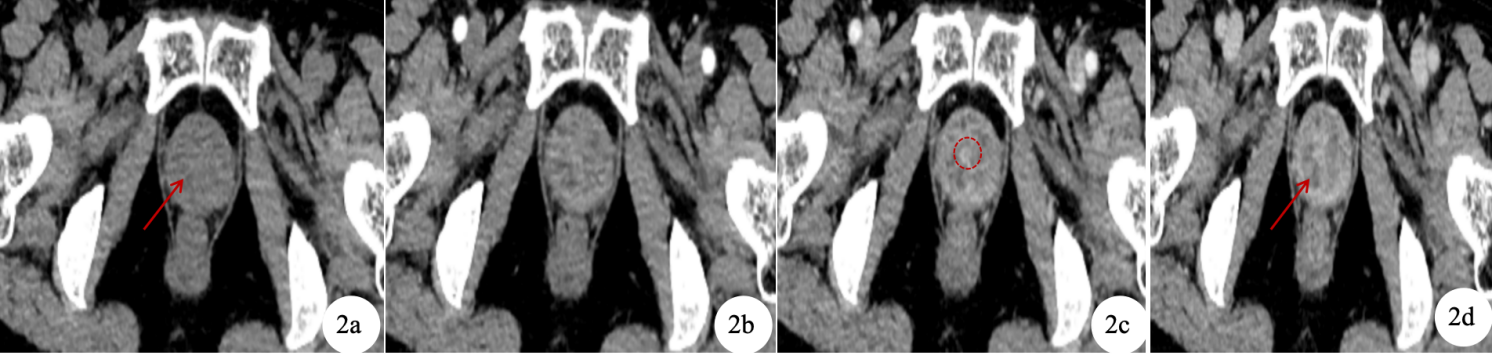
**

.**Supplementary Figure 2.**CT images of clear cell adenocarcinoma of the urethra (CCAU)****

**(a) Non-contrast CT: A soft tissue density mass is observed in the posteroinferior urethral region to the bladder, with partially ill-defined margins. Patchy areas of low density (red arrow) are noted within the mass. (b, c, d)Contrast-enhanced CT, arterial, venous, and delayed phases: The mass demonstrates marked heterogeneous enhancement. Patchy non-enhancing necrotic areas (red arrow) and a circumferentially enhancing urethral image (red dashed circle) are visible within the mass.**


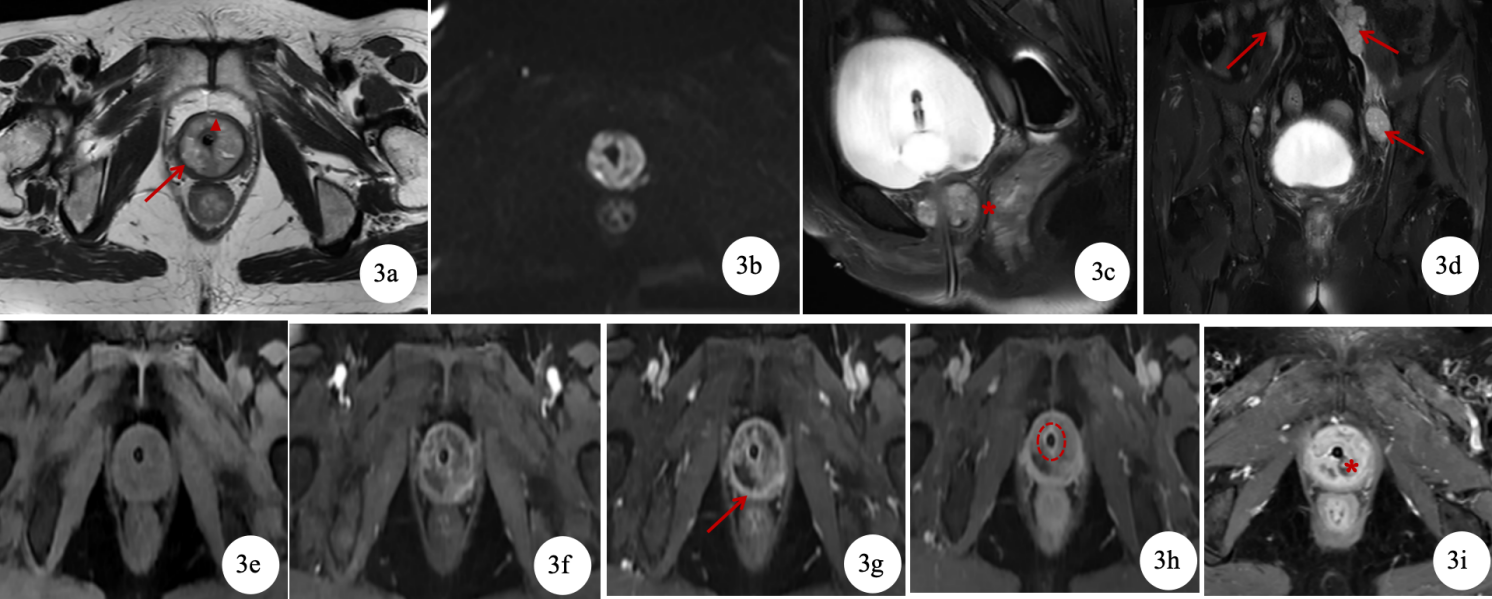


**Supplementary Figure 3.**MRI findings of clear cell adenocarcinoma of the urethra (CCAU)****

**(a) Axial T2WI: A annular, round-like mass is visible around the upper urethra, demonstrating slightly high signal intensity (compared to muscle). Within the mass, small patchy areas of high signal and punctate low signal foci (red triangle) are observed, surrounded by a low-signal capsule (red arrow) with well-defined margins. (b) DWI, b=1000 s/mm²: The mass shows restricted diffusion, with an ADC value of 0.79×10⁻³ mm²/s. (c)Sagittal T2WI: The mass exhibits clear boundaries with the bladder and vagina (red star), with posterior displacement of the vaginal wall. (d)Coronal T2WI: Multiple enlarged lymph nodes are visible along the bilateral iliac vessels (red arrow). (e)Pre-contrast mask: The mass shows iso- to slightly low signal intensity (compared to muscle).(f-i)DCE series: The tumor is hypervascular and demonstrates progressive heterogeneous enhancement. Non-enhanced necrotic areas are noted within the mass (red triangle), with prominent capsular enhancement (red arrow). Circumferential enhancement of the urethral wall is visible on certain sequences (red dashed circle), and the local urethral morphology appears irregular (red star).**

**
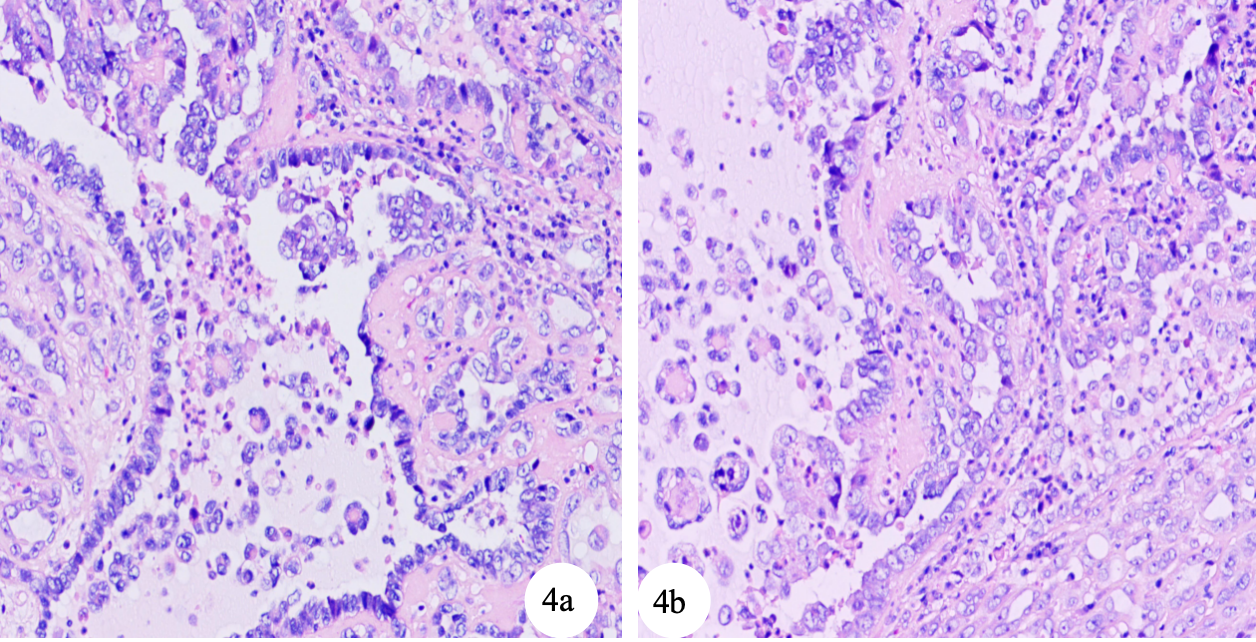
**

**Supplementary Figure4.**Histopathological features of clear cell adenocarcinoma of the urethra (CCAU)****

**(a, b) H&E staining, ×400: Tumor cells exhibiting clear cytoplasm and prominent nucleoli are observed.**

**
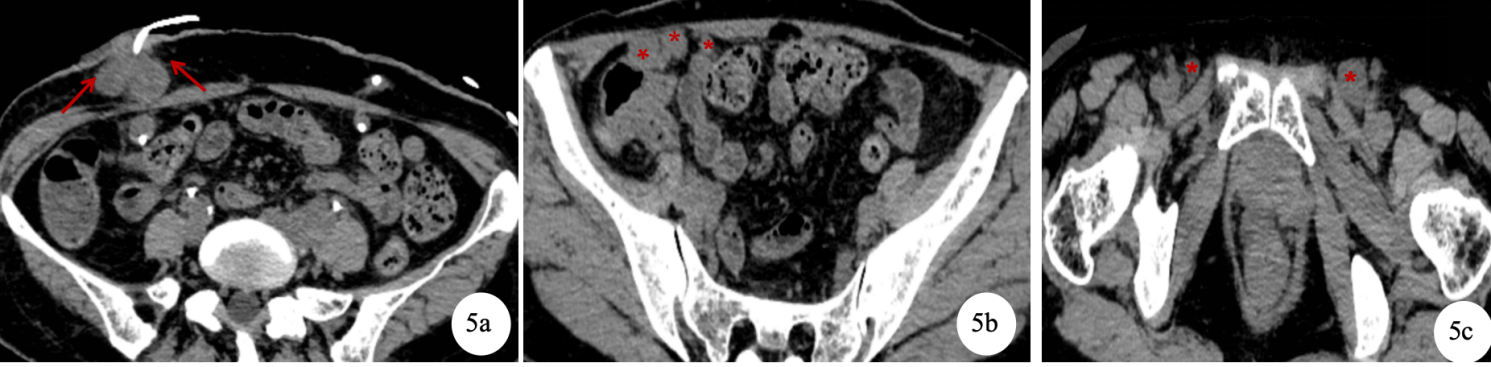
**

**Supplementary Figure 5.**CT images obtained 7 months postoperatively in a patient with CCAU****

**(a, b, c )Non-contrast CT: Demonstrate a soft tissue mass adjacent to the right ureteral abdominal wall stoma (red arrow), along with enlarged lymph nodes in the pelvic cavity and bilateral inguinal regions (red star).**
